# LEVERAGING AN “ETHICAL BY DESIGN” APPROACH TO CREATE MORE INHERENTLY RESPONSIBLE TECHNOLOGY TO SUPPORT AGING

**DOI:** 10.1093/geroni/igad104.1039

**Published:** 2023-12-21

**Authors:** Jennifer Boger

**Affiliations:** University of Waterloo, Waterloo, Ontario, Canada

## Abstract

Responsible technologies are technologies that appropriately support the needs, abilities, and values of the people using them. While many aspects of responsible technology are ubiquitous, there are a multitude of considerations that are specific to older adults. These age-related aspects must be properly understood and incorporated into the technology development and selection process if technology is to be inclusive of and useful to older adults. However, as many of these aspects are subjective, qualitative, and/or dynamic, developers often consider them to be too abstract, undefined, or difficult to address, resulting in their omission from the technology creation process. In this presentation we will discuss how we can leverage the concept of ‘Ethical by Design’ to drive the change in thinking and doing that is required to enable developers to engage with age-related concepts in technology design, development, and evaluation. Concepts such as user-centered design, values-based engineering, brave spaces, and co-creation will be presented as some of the ways we can directly access lived experiences and translate them into the creation of technologies that are more inclusive of older adults.

